# Exposure to polychlorinated biphenyls and organochlorine pesticides and risk of dementia, Alzheimer’s disease and cognitive decline in an older population: a prospective analysis from the Canadian Study of Health and Aging

**DOI:** 10.1186/s12940-019-0494-2

**Published:** 2019-06-14

**Authors:** Thierry Comlan Marc Medehouenou, Pierre Ayotte, Pierre-Hugues Carmichael, Edeltraut Kröger, René Verreault, Joan Lindsay, Éric Dewailly, Suzanne L. Tyas, Alexandre Bureau, Danielle Laurin

**Affiliations:** 1 Département de Génie d’imagerie médicale et de radiobiologie, École Polytechnique d’Abomey-Calavi, University of Abomey-Calavi, P.O. Box 2009, Cotonou, Abomey-Calavi, Republic of Benin; 2 Centre d’excellence sur le vieillissement de Québec, CHU de Québec-Université Laval Research Center, and Centre de recherche sur les soins et les services de première ligne de l’Université Laval, Québec, Canada; 3 Faculty of Pharmacy, Laval University, Québec, Canada; 4 Département de médecine sociale et préventive, Faculty of Medicine, Laval University, Québec, Canada; 5 Axe Santé des populations et pratiques optimales en santé, CHU de Québec-Université Laval Research Center, Québec, Canada; 6 Laboratoire de toxicologie, Institut national de santé publique du Québec, Québec, Canada; 7 Institut sur le vieillissement et la participation sociale des aînés, Université Laval, Québec, Canada; 8 Department of Epidemiology and Community Medicine, Faculty of Medicine, University of Ottawa, Ottawa, Canada; 9 School of Public Health and Health Systems, and Department of Psychology, University of Waterloo, Waterloo, Canada; 10 Centre de recherche de l’Institut universitaire en santé mentale de Québec, Québec, Canada

**Keywords:** Alzheimer’s disease, Dementia, Cognitive decline, Organochlorine pesticide, Polychlorinated biphenyl

## Abstract

**Background:**

Little attention has been paid to neurotoxicants on the risk of dementia. Exposure to known neurotoxicants such as polychlorinated biphenyls (PCBs) and organochlorine (OC) pesticides is suspected to have adverse cognitive effects in older populations.

**Objective:**

To assess whether plasma concentrations of PCBs and OC pesticides are associated with the risk of cognitive decline, Alzheimer’s disease (AD) and of all-cause dementia in the Canadian older population.

**Methods:**

Analyses were based on data from the Canadian Study of Health and Aging, a 3-phase, 10-year population-based study of individuals aged 65+ years. Analyses included 669 clinically assessed subjects, of which 156 developed dementia including 108 incident cases of AD. Subjects were screened at each phase with the 100-point Modified Mini-Mental State Examination (3MS), a measurement of global cognitive function. Statistical analyses included Cox proportional hazards model when the outcome was dementia or AD, and a repeated-measure mixed model when the outcome was the 3MS score.

**Results:**

No association of PCB and OC pesticides with the risk of dementia and AD was observed. Elevated concentrations of PCB congeners nos 118, 153, 156, 163, and OC pesticides 1,1,1-trichloro-2,2-bis(p-chlorophenyl)ethane (*p,p’*-DDT) and its metabolite 1,1-dichloro-2,2-bis(p-chlorophenyl)ethylene (*p,p’*-DDE) were significantly associated with cognitive decline as assessed with the 3MS. A posteriori analyses suggested that only *p,p’*-DDE was significantly related to a higher cognitive decline in time based on the 3MS among incident cases of dementia compared to subjects remaining nondemented.

**Conclusion:**

PCB and OC pesticide plasma concentrations were not related to the incident diagnosis of neither dementia, nor AD. Using the 3MS scores as the outcome, higher concentrations of four PCB congeners and two OC pesticides were associated with lower cognitive performances in subjects. The association of *p,p’*-DDE with cognitive decline in time in incident cases of dementia merits further investigation.

**Electronic supplementary material:**

The online version of this article (10.1186/s12940-019-0494-2) contains supplementary material, which is available to authorized users.

## Background

There is increasing evidence suggesting the role of environmental factors in the development of dementia or Alzheimer’s disease (AD) [1–3]. One systematic review including a meta-analysis reported a positive association between pesticide exposure and AD [4]. Since polychlorinated biphenyls (PCBs) and organochlorine (OCs) pesticides, also known as persistent OC compounds, were reported to induce cognitive, motor and behavioral deficits in animal models through a number of potential modes of action [5, 6], growing interest in ascertaining their relationship with cognitive impairment [7–11], all-cause dementia and AD [12–15] and Lewy pathology [16] has been mostly noticed in recent years. These few studies indicate a potential association between OC compound exposure and cognitive impairment and dementia, but the results are still inconsistent.

Using biomarkers of exposure to PCBs and OC pesticides, we previously found no significant association of elevated plasma concentrations of PCBs with the prevalence of all-cause dementia or AD in subjects from the Canadian Study of Health and Aging (CSHA) [13]. Elevated concentrations of some OC pesticides were significantly associated with a reduced prevalence of all-cause dementia, and hexachlorobenzene (HCB) with a reduced prevalence of AD. These findings contrast with those reported in a case-control study, where elevated serum 1,1-dichloro-2,2-bis(p-chlorophenyl)ethylene (*p,p’*-DDE) concentrations were associated with an increased risk for AD, especially in apolipoprotein E allele e4 (ApoE4) carriers [14].

The present study extends our previous findings in a more rigorous prospective study design to determine whether plasma PCB and OC pesticide concentrations are associated with the incidence of all-cause dementia, AD and cognitive decline. The potential modifying effects of sex, ApoE4 and total blood mercury concentrations were also investigated.

## Methods

### Study population

The CSHA is a national cohort study of dementia in older Canadians. Eighteen research centers across the country were involved. Methodological details have been described elsewhere [17, 18]. The baseline examination was carried out in 1991–1992 (CSHA-1) with two follow-ups. Each phase received approval from institutional ethics committees in participating centers. Subjects and/or family representatives gave written consent at each phase.

In CSHA-1, a random sample of 10,263 men and women, representative of the Canadian population aged 65 and over was drawn from the Enumeration Composite in Ontario and from Medicare lists in the other provinces for 36 urban and surrounding rural areas. Institutionalized participants were randomly selected from residents in stratified random samples of institutions in each region. The study excluded Yukon and the Northwest Territories, Indian reserves and military units. Of the study subjects, 9008 were living in the community, and 1255 in institutions.

Community-dwelling participants were screened for dementia using a cut-off of 77/78 on the 100-point Modified Mini-Mental State (3MS) examination [18]. Subjects who screened positive (3MS < 78), a random sample of those who screened negative and all institutionalized subjects were invited to attend an extensive standardized clinical evaluation. Of note, subjects who screened negative were invited to the clinical evaluation as part of other sub-studies in CSHA including validation or case-control studies [17].

A nurse (re)administered the 3MS, collected information on medication, and obtained the subject’s medical and family histories from a relative. A physician performed a standardized clinical and neurological examination. Non-fasting blood samples were drawn at the end of the examination for laboratory tests (required if dementia or delirium presumed) or collected for future analyses (optional). Finally, a psychometrist administered a neuropsychological test battery to subjects with a 3MS of 50 and over; results were interpreted by a neuropsychologist.

The physician and the neuropsychologist made independent preliminary diagnoses. Then, they reached a consensus diagnosis in a case conference according to Diagnostic and Statistical Manual of Mental Disorders, 3rd edition, revised criteria (DSM-III-R) for dementia [19]. Consensus diagnoses also included: no cognitive impairment; cognitive impairment with no dementia (CIND) according to DSM-III-R and the International Classification of Diseases, 10th revision criteria [20]; AD according to the National Institute of Neurological and Communicative Disorders and Stroke-Alzheimer’s Disease and Related Disorders Association criteria [21]; vascular dementia according to the International Classification of Diseases, 10th revision criteria; and other specific and unclassifiable dementia.

Information on risk factors was collected at baseline with a self-administered risk factor questionnaire covering socio-demographic characteristics, lifestyle, family and medical histories.

All subjects initially evaluated were contacted in 1996–1997 (CSHA-2) to assess changes in health status and functioning. The diagnostic process used in CSHA-2 was similar to the one used in CSHA-1.

Subjects clinically examined at CSHA-1 were automatically invited to the clinical examination. CSHA-2 diagnoses were made without knowledge of CSHA-1 diagnoses. Two final diagnoses were made: one according to CSHA-1 criteria; the other one, according to more recent criteria from the fourth edition of the DSM [22] for dementia and AD, and the National Institute of Neurological Disorders and Stroke-Association Internationale pour la Recherche et l’Enseignement en Neurosciences criteria [23] for vascular dementia.

The last phase of CSHA (CSHA-3) took place in 2001–2002. The 3MS cut-off was increased to 89/90 to focus on the progression of cognitive impairment. Subjects unable to complete the neuropsychological evaluation or with a diagnosis of CIND, or dementia according to the neuropsychologist were asked to attend the clinical examination. The final diagnoses were made according to the same processes and diagnostic criteria as those used in CSHA-2 [17].

### Study and biospecimen samples

**Fig. 1:**
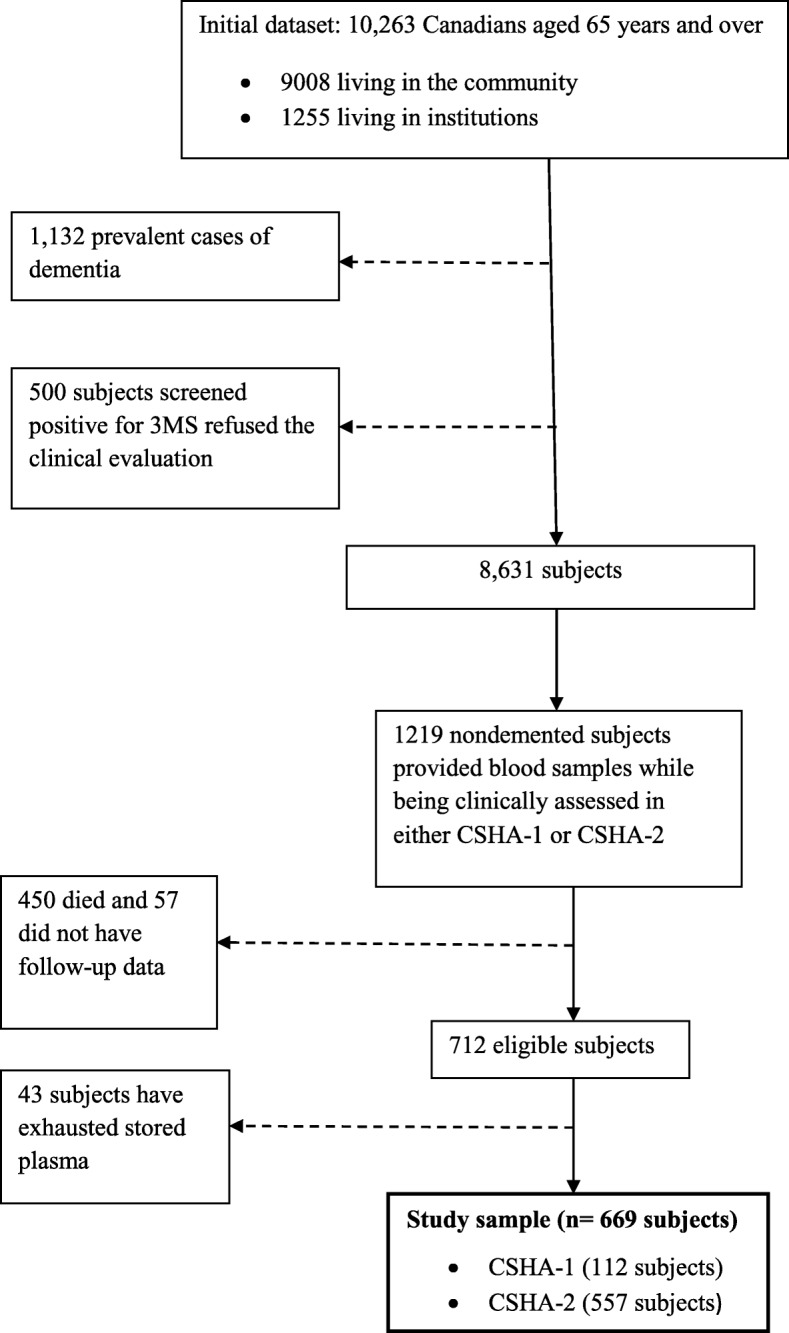
Flowchart of study sample

To be included in the analytic sample, subjects had to have their blood sample drawn while they were still cognitively normal, prior to a subsequent clinical evaluation, thus preserving the prospective nature of the data collection. Of 10,263 subjects, there were 1132 prevalent cases of dementia and 500 subjects who screened positive but refused the clinical evaluation, which left 8631 subjects. Of these, 1219 nondemented subjects provided blood samples while being clinically assessed in either CSHA-1 or CSHA-2; 450 died and 57 did not have follow-up data, which left 712 eligible subjects. Of these, 43 subjects had to be excluded because of exhausted stored plasma, leaving 669 subjects for statistical analyses including 642 with whole blood samples (Fig. 1). Study entry was defined as the moment of providing blood (CSHA-1 for 112 subjects and CSHA-2 for 557 subjects). There was no difference concerning age at CSHA baseline and sex between subjects in the analytic sample (*n* = 669) and those from the CSHA cohort who could not be included (*n* = 7954). Subjects from the analytic sample had slightly fewer years of education than those not included (mean ± SD, 9.8 ± 4.1 and 10.3 ± 3.8 years, respectively; *p* < 0.01).

### Laboratory assessment

Plasma and ethylenediaminetetraacetic acid (EDTA, added as an anticoagulant) whole-blood samples from participants were stored at the National Microbiology Laboratory, Winnipeg, Canada. Plasma OC compound concentrations were determined as described previously [24] at the *Laboratoire de toxicologie* of the *Institut national de santé publique du Québec* (INSPQ), Quebec, Canada, which is accredited under ISO 17025 by the Standards Council of Canada [25]. Briefly, 15 PCB congeners numbers 28, 52, 99, 101, 105, 118, 128, 138, 153, 156, 163, 170, 180, 183, 187 and 11 OC pesticides or their metabolites [aldrin, mirex, α-chlordane, γ-chlordane, oxychlordane, *cis*-nonachlor, *trans*-nonachlor, β-hexachlorocyclohexane (β-HCH), hexachlorobenzene (HCB), 1,1,1-trichloro-2,2-bis(*p*-chlorophenyl)ethane (*p,p’*-DDT), 1,1-dichloro-2,2-bis(*p*-chlorophenyl)ethylene (*p,p’*-DDE)] were identified and quantified by gas chromatography-mass spectrometry using negative chemical ionisation. Limits of detection (LOD) ranged from 0.01 to 0.70 μg/L (PCB congeners), and from 0.005 to 0.20 μg/L (OC pesticides/metabolites). Between day coefficients of variation were ≤ 11% for PCB congeners except for PCB 28 (13.3%) and PCB 128 (20.8%), and ≤ 12% for OC pesticides except for oxychlordane (14.7%), *p,p’*-DDT (16.9%) and aldrin (29.7%).

Copper, lead, mercury and zinc were also determined at the INSPQ in EDTA whole-blood samples by inductively coupled plasma mass spectrometry (ICP-MS). Blood samples are diluted in ammonium hydroxide and metals are brought to their elementary form by passing through argon plasma before being identified and quantified by mass spectrometry. Lead, copper and zinc were detected in all samples. Mercury was not detected in 39 out of 642 samples (LOD = 0.5 nmol/L). Between-day coefficients of variation were 3.0% for copper, 2.7% for lead, 3.8% for mercury, and 3.9% for zinc.

Cholesterol and triglycerides concentrations were determined by enzymatic tests on Roche automated clinical chemistry analyzers at the *Laboratoire de biochimie du Centre de recherche du CHU de Québec*, Québec, Canada. Total lipids were calculated using the two-component formula of Phillips et al. [26]. Apolipoprotein E genotypes were determined by the modified method of Zivelin and colleagues [27].

The measurement of biomarkers was approved by the ethic committee of the CHU de Québec, Québec, Canada.

### Covariates and potential confounders

Information on covariates and potential confounders were extracted from the risk factor questionnaire at baseline or the clinical examination at blood collection. These included age (years), education (years), sex, body mass index (BMI, kg/m^2^), the presence of ApoE4 (yes/no) and residence area (rural/urban). Smoking status was categorized as ever been smoking regularly (almost every day) or not, and alcohol intake, as ever been drinking regularly (at least once a week) or not. A vascular score was defined as a summation score (0–3) for three vascular risk factors including hypertension (defined as a supine blood pressure > 160 mmHg systolic or 95 mmHg diastolic, or a physician’s diagnostic or the use of medication for hypertension), history of myocardial infarction or stroke, and history of diabetes mellitus.

### Statistical analysis

Analyses were performed on individual OC compounds with at least 60% of samples with measurements above the LOD [13]; these included PCB congeners 105, 118, 138, 153, 156, 163, 170, 180, 183 and 187, and OC pesticides β-HCH, HCB, oxychlordane, *trans*-nonachlor, *cis*-nonachlor, *p,p’*-DDE, and *p,p’*-DDT. Samples with measures below detection were set to one-half LOD of each analyte [28]. Baseline characteristics of demented and nondemented subjects were compared using chi-square tests for categorical variables and Student’s *t* tests or Wilcoxon rank-sum tests for continuous variables. Concentrations of PCBs, OC pesticides and metals except copper were skewed to the right and log-transformed. To take into account observations with missing covariates, multiple imputation (MI) was also performed [29]. Five imputed datasets were generated and individually analysed as complete data before being pooled. Type 3 tests were pooled using the methodology presented in van Ginkel and Kroonenberg’s paper [30].

Hazard ratios (HRs) with 95% confidence intervals (CIs) were calculated using Cox proportional hazards model with delayed entry and age as the time scale to assess the relationship between PCBs and OC pesticides and the incidence of all-cause dementia or AD. Since onset of disease is estimated to occur between two subsequent evaluations, time-to-event on the age scale was interval-censored between the two evaluations. Subjects who remained cognitively normal or died were censored at the time of last evaluation. The proportional hazards assumptions were tested [31] and found to be satisfied. The linearity assumption for continuous variables was found to be satisfied [32]. Two models were built. Since lipid-standardized OC concentrations (i.e. OC concentrations divided by total plasma lipid concentrations) are highly prone to bias [33, 34], and that total plasma lipids may be related to dementia or AD, total plasma lipid concentrations were rather included in both models as a separate covariate. A first model was adjusted for total lipids, age as scale time, sex, education, and ApoE4. The second model was additionally adjusted for BMI, smoking status, alcohol intake, residence area, vascular score, copper, lead, mercury and zinc. Lead and mercury are considered as suspected neurotoxicants [1]; serum copper and zinc concentrations might be considered as potential markers for AD [35]. PCBs, OC pesticides, total plasma lipids, education, BMI, vascular score, copper, lead, mercury and zinc were treated as continuous variables and introduced in the model on usual linear scale.

A repeated-measures mixed model was used to assess the relationship of PCBs and OC pesticides on cognitive decline as measured with the 3MS. This analysis had the advantage of increasing statistical power given that the observations were subjects’ 3MS scores at each clinical examination, but to the detriment of the sensibility of the measure of the cognitive status. The time variable was defined by the 3 phases of CSHA. A first model was adjusted for total lipids, CSHA phase as well as age, sex, education, and ApoE4. The second model was additionally adjusted for BMI, smoking status, alcohol intake, residence area, vascular score, copper, lead, mercury and zinc.

The effect modification by sex, ApoE4 and total mercury [36] was tested on the associations of four indicator PCBs (congeners 118, 138, 153, 180) and three sentinel OC pesticides (β-HCH, *trans*-nonachlor, *p,p’*-DDE) with dementia, AD and cognitive decline by entering interaction terms in fully-adjusted models. These OC compounds are characterized by both high prevalence (i.e. 98% of subjects had concentrations above LODs) and high concentrations [24].

A 2-tailed *p*-value < 0.05 was considered to indicate statistical significance. Analyses were performed using SAS software (version 9.3 SAS Institute Inc., Cary, NC, USA).

## Results

Over a mean follow-up of 5.1 ± 0.3 years, 513 subjects remained free of dementia and 156 developed dementia, including 108 cases of AD. Compared with cognitively normal subjects, incident cases of dementia were significantly older (82.4 vs. 80.1 years), had fewer years of education (9.0 vs. 10.1 years), showed a higher proportion of ApoE4 (25.2 vs. 18.0%) and had lower concentrations of mercury (Table 1). There was no difference for sex, BMI, smoking status, alcohol drinking, residence area, vascular score, and concentrations of copper, lead and zinc between the two groups. OC concentrations were not different between the two groups (Table 2). Plasma concentrations of PCB congeners and OC pesticides were not associated with the risk of dementia or AD in any models (Table 3).

**Table 1:** Selected characteristics from the analytic study sample (*n* = 669)

Characteristics	Non-demented individuals (*n* = 513)	Incident cases of dementia (*n* = 156)	*P-*value
Age at study entry, y	80.1 ± 6.12	82.4 ± 6.60	< 0.001
Sex, female, n (%)	304 (59.3)	102 (65.4)	0.17
Education, y	10.1 ± 4.1	9.0 ± 4.1	< 0.01
BMI, kg/m^2^	25.9 ± 4.6	25.4 ± 5.2	0.30
Total lipids, g/L	6.1 ± 1.7	6.0 ± 2.0	0.63
ApoE4 carrier, n (%)	92 (18.0)	39 (25.2)	< 0.05
3MS score at study entry	88 ± 8.4	83 ± 9.8	< 0.001
Residence area, n (% urban)	448 (87.8)	139 (90.3)	0.41
Smoking, n (% yes) ^a^	217 (46.7)	67 (48.6)	0.70
Alcohol drinking, n (% yes)^b^	176 (37.7)	44 (32.4)	0.26
Vascular score	1.19 ± 0.83	1.26 ± 0.88	0.38
Blood heavy metal concentrations			
Copper, median (IQR), μmol/L	13.0 (12.0–14.0)	13.0 (12.0–14.0)	0.15
Lead, median (IQR), μmol/L	0.15 (0.11–0.20)	0.14 (0.10–0.20)	0.55
Mercury, median (IQR), nmol/L	3.3 (1.7–5.9)	2.4 (1.1–4.6)	< 0.001
Zinc, median (IQR), μmol/L	340 (270–460)	345 (270–455)	0.88

Note: *BMI* body mass index, *IQR* interquartile range

Study entry was set at the moment of blood collection (*n* = 112 at CSHA-1 and *n* = 557 at CSHA-2)

Values are represented as mean ± standard deviation unless mentioned otherwise

*P*-values were obtained using χ^2^ test for dichotomous variables and *t*-tests or non-parametric Wilcoxon rank-sum tests for continuous variables, as applicable

^a^ Indicates ever been smoking regularly (nearly everyday)

^b^ Indicates ever been drinking regularly (once a week)

Information was missing for 35 subjects on BMI (21 for nondemented and 14 for demented subjects); 3 subjects on ApoE4 status (2 for nondemented vs. 1 for demented subjects); 5 subjects on residence area (3 for nondemented vs. 2 for demented subjects); 66 subjects on smoking (48 for nondemented vs. 18 for demented subjects); 66 subjects on alcohol drinking (46 for nondemented vs. 20 for demented subjects); 33 subjects on plasma total lipids (27 for nondemented vs. 6 for demented subjects)

**Table 2:** Median plasma PCB and OC pesticide concentrations (μg/L) (*n* = 669)

OC concentrations	Non-demented individuals (*n* = 513)	Incident cases of dementia (*n* = 156)	*P*-value
PCB congeners			
PCB 105	0.03 (0.01–0.04)	0.03 (0.02–0.04)	0.82
PCB 118	0.14 (0.08–0.23)	0.14 (0.09–0.23)	0.85
PCB 138	0.24 (0.17–0.36)	0.24 (0.16–0.35)	0.77
PCB 153	0.43 (0.30–0.60)	0.44 (0.28–0.61)	0.98
PCB 156	0.06 (0.04–0.08)	0.06 (0.04–0.08)	0.34
PCB 163	0.08 (0.05–0.11)	0.08 (0.05–0.12)	0.43
PCB 170	0.10 (0.07–0.14)	0.10 (0.07–0.15)	0.63
PCB 180	0.34 (0.24–0.49)	0.35 (0.24–0.51)	0.82
PCB 183	0.03 (0.02–0.05)	0.03 (0.02–0.05)	0.62
PCB 187	0.10 (0.06–0.14)	0.09 (0.06–0.14)	0.52
OC pesticides			
β-HCH	0.12 (0.08–0.19)	0.13 (0.08–0.19)	0.88
HCB	0.17 (0.10–0.27)	0.19 (0.10–0.30)	0.28
Oxychlordane	0.11 (0.07–0.14)	0.11 (0.08–0.14)	0.87
*cis*-Nonachlor	0.02 (0.01–0.03)	0.02 (0.01–0.03)	0.37
*trans*-Nonachlor	0.15 (0.10–0.21)	0.14 (0.11–0.20)	0.77
*p,p’*-DDT	0.07 (0.02–0.13)	0.07 (0.02–0.13)	0.80
*p,p’*-DDE	4.10 (2.10–7.60)	4.10 (2.00–8.05)	0.82

Note: *β-HCH* beta hexachlorocyclohexane, *HCB* hexaclorobenzene, *OC* organochlorine compound, *PCBs* polychlorinated biphenyl, *p,p’*-DDT, 1,1,1-trichloro-2,2-bis(*p*-chlorophenyl)ethane, *p,p’*-DDE, 1,1-dichloro-2,2-bis(*p*-chlorophenyl)ethylene

Values in parentheses represent the interquartile range

*P*-values were obtained using non-parametric Wilcoxon rank-sum tests

**Table 3:** HRs for all-cause dementia and AD per 1-unit log increase in plasma PCB and OC pesticide concentrations (μg/L)

OC	All-cause dementia (*n* = 669)		Alzheimer’s disease (*n* = 621)	
	Model 1	Model 2	Model 1	Model 2
	HR (95%CI)	HR (95% CI)	HR (95% CI)	HR (95% CI)
PCB congeners				
PCB 105	0.93 (0.76, 1.13)	0.95 (0.77, 1.17)	0.91 (0.71, 1.17)	0.97 (0.75, 1.26)
PCB 118	0.94 (0.75, 1.18)	0.97 (0.77, 1.23)	0.93 (0.71, 1.23)	1.00 (0.75, 1.32)
PCB 138	0.92 (0.73, 1.16)	0.95 (0.76, 1.20)	0.96 (0.71, 1.29)	0.99 (0.74, 1.34)
PCB 153	0.95 (0.73, 1.24)	1.00 (0.77, 1.31)	0.94 (0.68, 1.31)	0.99 (0.71, 1.38)
PCB 156	1.16 (0.87, 1.55)	1.23 (0.91, 1.65)	1.09 (0.76,1.54)	1.14 (0.79, 1.65)
PCB 163	1.03 (0.79, 1.34)	1.09 (0.83, 1.42)	1.01 (0.73, 1.40)	1.08 (0.77, 1.50)
PCB 170	1.07 (0.83, 1.37)	1.11 (0.87, 1.43)	1.03 (0.76, 1.40)	1.08 (0.78, 1.48)
PCB 180	1.02 (0.80, 1.31)	1.07 (0.84, 1.37)	0.98 (0.73, 1.33)	1.03 (0.75, 1.41)
PCB 183	0.90 (0.72, 1.13)	0.94 (0.75, 1.17)	0.91 (0.69, 1.21)	0.94 (0.70, 1.25)
PCB 187	0.93 (0.75, 1.16)	0.97 (0.78, 1.22)	0.90 (0.68, 1.18)	0.95 (0.72, 1.26)
OC pesticides				
β-HCH	1.17 (0.95, 1.46)	1.18 (0.95, 1.47)	1.20 (0.90, 1.58)	1.16 (0.88, 1.54)
HCB	0.97 (0.79, 1.20)	1.01 (0.81, 1.25)	1.06 (0.81, 1.38)	1.14 (0.87, 1.50)
Oxychlordane	1.07 (0.80, 1.43)	1.13 (0.83, 1.53)	1.22 (0.85, 1.76)	1.33 (0.91, 1.94)
*cis*-Nonachlor	0.94 (0.75, 1.17)	0.99 (0.79, 1.24)	0.95 (0.73, 1.24)	1.04 (0.79, 1.38)
*trans*-Nonachlor	0.98 (0.74, 1.28)	1.03 (0.78, 1.36)	1.02 (0.73, 1.43)	1.10 (0.78, 1.55)
*p,p’*-DDT	1.03 (0.86, 1.23)	1.04 (0.87, 1.26)	1.02 (0.81, 1.28)	1.08 (0.86, 1.36)
*p,p’*-DDE	0.94 (0.81, 1.10)	0.96 (0.82, 1.12)	0.99 (0.81, 1.21)	1.01 (0.82, 1.23)

Note: *AD* Alzheimer’s disease, *BMI* body mass index, *β-HCH* beta hexachlorocyclohexane, *HCB* hexaclorobenzene, *OC* organochlorine, *HR* Hazards ratio, *PCBs* polychlorinated biphenyl, *p,p’*-DDT, 1,1,1-trichloro-2,2-bis(*p*-chlorophenyl)ethane, *p,p’*-DDE, 1,1-dichloro-2,2-bis(*p*-chlorophenyl)ethylene

Model 1 was adjusted for total lipids and age as time scale, sex, education, and ApoE4

Model 2 was additionally adjusted for BMI, smoking, alcohol drinking, residence area, vascular score, mercury, lead, cooper and zinc

**Fig. 2:**
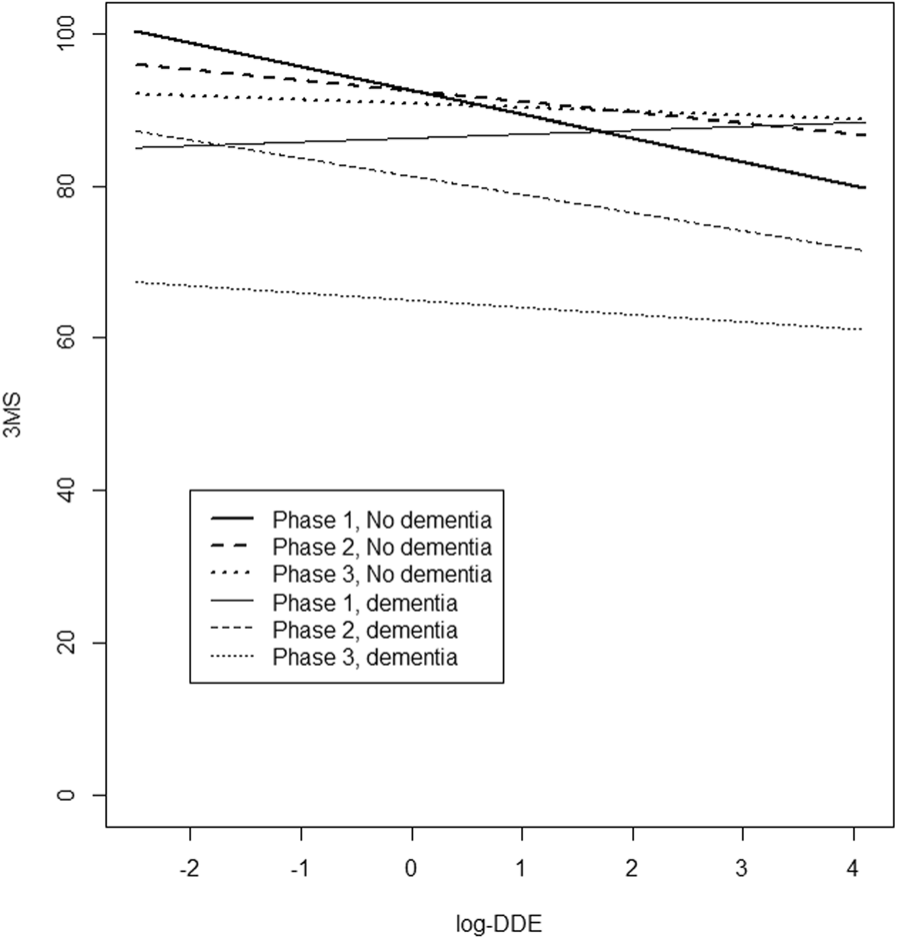
Relationship between 3MS at each of three CSHA phases and log-DDE according to dementia incidence

Table 4 summarizes the beta coefficients associated with individual OC compound concentrations in relation to 3MS scores. As mentioned, no significant interaction of OC compounds and time was detected, which means that OC compounds might have affected study entry cognitive performance, but did not accelerate cognitive decline. In the first model, the log-transformed concentrations of PCB congeners nos. 118, 153, 156, 163 and OC pesticide *p,p’*-DDT and its main metabolite (*p,p’*-DDE) were significantly associated with lower 3MS scores.. All these associations remained significant in the second model. We further investigated these specific results by entering in the models a triple interaction term including the presence of an incident diagnosis of dementia, CSHA period and, PCB or pesticide concentrations. These a posteriori analyses showed that only *p,p’*-DDE was significantly associated with dementia-related cognitive decline (*p* = 0.03). Figure 2 shows the estimated relationship between the 3MS and the *p,p’*-DDE concentrations on the log-scale for each CSHA phase according to dementia incidence. For incident cases of dementia, we see that the 3MS scores decreased at each phase, and that *p,p’*-DDE concentrations were associated with lower 3MS scores in CSHA-2 and -3, but not in CSHA-1. As for subjects remaining nondemented, we see little differences in the relationship between *p,p’*-DDE concentrations and 3MS scores at each CSHA phase. No association was found between the 3MS and PCBs 105, 138, 170, 180, 183, 187, and OC pesticides β-HCH, HCB, oxychlordane, *cis*-nonachlor and *trans*-nonachlor.

**Table 4:** Estimated coefficients of plasma log-transformed PCB and OC pesticide concentrations (μg/L) in relation to 3MS scores

OC concentrations	Model 1			Model 2		
	β	SE	*P-value*	β	SE	*P-value*
PCB congeners						
PCB 105	−0.811	0.509	0.111	−0.780	0.544	0.151
PCB 118	−1.225	0.581	0.035	−1.207	0.611	0.048
PCB 138	−1.219	0.626	0.052	−1.223	0.644	0.058
PCB 153	−1.387	0.705	0.049	− 1.449	0.727	0.046
PCB 156	−1.509	0.755	0.046	−1.586	0.780	0.042
PCB 163	−1.501	0.699	0.032	−1.602	0.724	0.027
PCB 170	−0.830	0.691	0.230	−0.908	0.708	0.200
PCB 180	−0.521	0.688	0.448	−0.611	0.707	0.390
PCB 183	−0.948	0.611	0.121	−0.968	0.629	0.124
PCB 187	−0.149	0.580	0.797	−0.215	0.601	0.721
OC pesticides						
β-HCH	−0.751	0.590	0.203	−0.699	0.606	0.249
HCB	−0.737	0.519	0.156	−0.644	0.541	0.233
Oxychlordane	−0.602	0.803	0.454	−0.540	0.824	0.512
*cis*-nonachlor	−0.044	0.585	0.940	+ 0.038	0.627	0.952
*trans*-Nonachlor	−0.351	0.714	0.623	−0.332	0.742	0.655
*p,p’*-DDT	−1.717	0.468	< 0.001	−1.693	0.487	< 0.001
*p,p’*-DDE	−1.554	0.414	< 0.001	−1.578	0.422	< 0.001

Note: *3MS* Modified Mini-Mental State Examination, *β* beta, *BMI* body mass index, *β-HCH* beta hexachlorocyclohexane, *HCB* hexaclorobenzene, *OC* organochlorine, *PCBs* polychlorinated biphenyls, *p,p’*-DDT, 1,1,1-trichloro-2,2-bis(*p*-chlorophenyl)ethane; *p,p’*-DDE, 1,1-dichloro-2,2-bis(*p*-chlorophenyl)ethylene, *SE* standard error

Repeated measures regression models with unstructured correlation matrix

Model 1 was adjusted for total lipids and CSHA phase, age, sex, education, and ApoE4

Model 2 was additionally adjusted for BMI, smoking, alcohol drinking, residence area, vascular score, copper, lead, mercury and zinc

No effect modification by sex, ApoE4 and total mercury was found in any regression analyses (data not shown).

## Discussion

Few observational studies have explored the potential association between exposure to organochlorines and risk of all-cause dementia, AD [12–15], and cognitive impairment [7–11] over the last years among seniors. The present study was designed to evaluate prospectively such associations among Canadians aged 65 and older.

Our study found no association between the exposure to PCBs and OC pesticides and the incidence of dementia or AD. However, analyses using the 3MS scores as the outcome showed that higher concentrations of PCB congeners nos. 118, 153, 156, 163 and OC pesticides (*p,p’*-DDT and its metabolite *p,p’*-DDE) were significantly associated with lower cognitive performances. A posteriori analyses suggested that only *p,p’*-DDE was significantly associated with dementia-related cognitive decline. An increase in the concentration of *p,p’*-DDE reduced 3MS scores more importantly over time in incident cases of dementia compared to nondemented subjects. However, these results were not strictly monotonous in time and may have been more susceptible to attrition in the last phase of CSHA, particularly for incident cases of dementia.

There is a paucity of research on the association of PCBs and OC pesticides with the risk of dementia in older populations. In addition to our study, two epidemiological studies specifically examined the association of PCBs and OC pesticides with the risk of dementia [12, 15]. Compared with the U.S. population, a retrospective mortality study of 17,321 PCB-exposed workers reported a two-fold mortality excess of dementia and AD among women in the high exposure group [15]. Limitations included the small numbers of death and the PCB exposure assessment method based on a modified job-exposure matrix. In an analysis from the Cache County Memory Study including 3084 participants, technical grade DDT was associated with a marginally increased risk of AD [12]. A limitation was the pesticide exposure assessment method based on in-person occupational history questionnaire that is often prone to recall bias. Finally, a case-control study including 86 cases of AD and 79 controls reported a significant association between elevated serum *p,p’*-DDE concentrations and an increased risk for AD in a US clinical population [14].

The present finding of no significant association of PCB congeners with incident cases of dementia and AD does not differ from the findings observed in our previous study with prevalent cases of dementia and AD [13], but the association of OC pesticides/metabolites does differ. In the previous study, elevated concentrations of HCB, *cis*-nonachlor and *p,p’*-DDT were associated with a reduced prevalence of dementia. HCB was also associated with a reduced prevalence of AD. These results are more fallacious than compelling ones as acknowledged in the paper*.* In the present study, none of the OC pesticides/metabolites were associated with the incidence of dementia and AD. A possible explanation for these discordant results might be that current results were obtained with incident cases of dementia which would attenuate the potential for reverse causality bias as compared to results obtained with prevalent cases*.*

The evidence supporting an association between exposure to OC pesticides and cognitive impairment in older adults is limited. Using the 3MS as a continuous outcome allowed us to test with greater statistical power the associations of PCBs and OC pesticides/metabolites with cognitive decline, which has been suggested to predict the development of dementia [37]. Increased concentrations of four PCBs (congeners 118, 153, 156, 163) and two OC pesticides (*p,p’*-DDT and its metabolite *p,p’*-DDE) were found to be significantly associated with a lower cognitive function. These findings are somewhat in line with previous studies which reported associations between a lower performance in some cognitive domains and exposure to PCBs [7, 11, 38–41] in population- or community-dwelling older subjects, to *p,p’*-DDT or/and *p,p’*-DDE in population-dwelling older subjects (US National Health and Nutrition Examination Survey, 1999–2002) [8, 9] and in retired malaria-control Costa-Rican workers [42]. In contrast to our study, neither *p,p’*-DDT nor *p,p’*-DDE were significantly associated with a lower cognitive function measured with the Mini-Mental State Examination (MMSE) among occupational OC pesticide exposure Costa Ricans [43]. Richardson et al. reported a significant association between elevated concentrations of serum *p,p’*-DDE and lower MMSE scores [14], especially in ApoE4 carriers. This interaction was not found in our sample, but the prevalence of ApoE4 carriers was much lower (20%) than theirs (global: 51%; 35% in controls and 65% in AD cases). Furthermore, mean concentrations of DDE in our sample varied between 1.33 ng/mg cholesterol in cognitively normal subjects and 1.57 ng/mg cholesterol in AD cases whereas those values varied respectively between 0.69 and 2.64 ng/mg cholesterol in the study of Richardson et al. [14].

In our analytic sample, despite that all subjects were clinically diagnosed nondemented at study entry, those who developed dementia had a lower mean 3MS score compared to those remaining nondemented (83 vs. 88, respectively *p* < 0.001). This suggests that the group of incident cases of dementia comprised at study entry a higher proportion of subjects who were in the prodromal stage of AD. Indeed, this group included a higher proportion of subjects with CIND compared to those remaining nondemented (65% vs. 23%). CIND has been found with the highest relative predictive power for the 5-year progression to dementia in CSHA [44].

Our analyses using the 3MS scores as the outcome may therefore be more informative. Not all PCBs and OC pesticides/metabolites that theoretically have a high potential to produce alterations in neurotransmitter systems in the brain are associated with lower cognitive performance. Except for PCBs 118 and 156, which belong to mono-ortho dioxin-like PCBs, other PCBs (congeners 153 and 163) also involved in the association with lower cognitive performances are non-dioxin-like PCBs. Our results indicate that different types of PCB congeners and OC pesticides/metabolites, with different structures and toxicologic properties may affect the central nervous system differently, inducing cognitive impairment over time in older subjects.

Subtle changes in central nervous system functions induced by chronic exposure to PCBs 118, 153, 156, 163 and OC pesticides such as *p,p’*-DDT and *p,p’*-DDE may have important repercussions on cognition in older subjects by reducing their capacity to compensate for age-related impairment. Disruption of Ca2+ homeostasis and of neurotransmitter release, excitotoxicity, formation of reactive oxygen species, oxidative stress, neuronal dysfunction and apoptosis have been proposed as potential mechanisms of action [6].

Some limitations in this study should be noted. The analyses were restricted to 669 clinically assessed subjects out of the eligible subjects. No difference between subjects from the analytic sample and those who could not be included in terms of age and sex at the beginning of study was noted, and the difference in years of education between the two groups was less than 1 year. The fact that we retained in the analytic sample only subjects with follow-up information may have produced biased results. However, when we compared the characteristics of excluded subjects with exposure data (*n* = 507) with those from the analytic sample (Additional file 1: Table S1), excluded subjects showed characteristics generally associated with an increased risk of dementia (older age, fewer years of education, lower 3MS score at entry), but no difference in plasma concentrations of PCB congeners and OC pesticides. Our results are based on a single measurement of PCBs and OC pesticides, but these compounds are recognized as stable toxicants with long half-lives (years) in humans, which reduces the possibility of exposure misclassification. Though the present study looks at individual compounds, novel modeling methods such as the Bayesian kernel machine regression [45] could be used to explore mixtures of OC compounds in larger samples, which may be more meaningful at the population level. However, it is not clear that our sample size allows for appropriate coverage of all possible mixtures of the 10 PCBs and 7 OC pesticides for such an analysis. Our results are also based on PCB concentrations lower than those from the 2001–2002 National Health and Nutrition Examination Surveys (NHANES), which may limit the possibility of finding an association. Finally, only three cases of Parkinson disease out of the 156 incident cases of dementia were observed which limits the possibility of confounding, but residual and unmeasured confounding remains possible.

## Conclusion

The present study suggests that exposure to PCBs and OC pesticides were not associated with the incidence of dementia and AD. In contrast, exposure to PCB congeners 118, 153, 156 and 163, and two OC pesticides, *p,p’*-DDT and *p,p’*-DDE, was associated with reduced mean cognitive performances. Of these, *p,p’*-DDE was associated with cognitive decline on the 3MS scores related to dementia. Further prospective research using these specific biomarkers of OC exposure, notably *p,p’*-DDE, in a larger sample of subjects clinically evaluated is warranted.

## Additional file

Additional file 1: **Table S1.** Characteristics of included (*n* = 669) and excluded (*n* = 507) subjects at study entry. (DOCX 42 kb)

## Data Availability

The data supporting the conclusions of this work are included within the manuscript and its Additional file 1: Table S1. Characteristics of included (*n* = 669) and excluded (*n* = 507) subjects at study entry). Further, the dataset analysed during the current study is available from the corresponding author on reasonable request.

## Abbreviations

3MS: Modified Mini-Mental State Examination
AD: Alzheimer’s disease
ApoE4: Apolipoprotein E allele e4
BMI: Body mass index
CI: Confidence intervals
CIHR: Canadian Institutes of Health Research
CIND: Cognitive impairment with no dementia
CSHA: Canadian Study of Health and Aging
DSM III-R: Diagnostic and Statistical Manual of Mental Disorders, 3rd edition, revised criteria
EDTA: Ethylenediaminetetraacetic acid
HCB: Hexachlorobenzene
HR: Hazard ratios
ICP-MS: Inductively coupled plasma mass spectrometry
INSPQ: Institut national de santé publique du Québec
IQR: Interquartile range
LOD: Limits of detection
MI: Multiple imputation
NHANES: National Health and Nutrition Examination Surveys
OC: Organochlorine
*p,p’*-DDE: 1,1-dichloro-2,2-bis(p-chlorophenyl)ethylene
*p,p’*-DDT: 1,1,1-trichloro-2,2-bis(p-chlorophenyl)ethane
PCB: Polychlorinated biphenyl
β-HCH: β-hexachlorocyclohexane
