# Supplementary material for: Exposure to polychlorinated biphenyls and organochlorine pesticides and risk of dementia, Alzheimer’s disease and cognitive decline in an older population: a prospective analysis from the Canadian Study of Health and Aging
Source: Environ Health. 2019 Jun 14;18:57. doi: 10.1186/s12940-019-0494-2 (PMC6570931; doi:10.1186/s12940-019-0494-2)
Supplement: Supplementary file 1 — Table S1. Characteristics of included (n = 669) and excluded (n = 507) subjects at study entry. (DOCX 42 kb) [file 12940_2019_494_MOESM1_ESM.docx]

**Supplementary Material**

**Exposure to Polychlorinated Biphenyls and Organochlorine Pesticides and Risk of Dementia, Alzheimer’s disease and Cognitive Decline in an Older Population: A Prospective Analysis from the Canadian Study of Health and Aging**

Thierry Comlan Marc Medehouenou,^1,2,3^ Pierre Ayotte,^4,5,6^ Pierre-Hugues Carmichael,^2^ Edeltraut Kröger,^2,3^ René Verreault,^2,4,5,7^ Joan Lindsay,^4,8^ Éric Dewailly,^4,5,6^^[[1]](#footnote-1)^ Suzanne L. Tyas,^9^ Alexandre Bureau,^4,10^  Danielle Laurin^2,3,5,7^

^1^ Département de Génie d’imagerie médicale et de radiobiologie, École Polytechnique d’Abomey-Calavi, University of Abomey-Calavi, Abomey-Calavi, Republic of Benin

^2^ Centre d’excellence sur le vieillissement de Québec, CHU de Québec-Université Laval Research Center, and Centre de recherche sur les soins et les services de première ligne de l’Université Laval, Québec, Canada

^3^ Faculty of Pharmacy, Laval University, Québec, Canada

^4^ Département de médecine sociale et préventive, Faculty of Medicine, Laval University, Québec, Canada

^5^ Axe Santé des populations et pratiques optimales en santé, CHU de Québec-Université Laval Research Center, Québec, Canada

^6^ Laboratoire de toxicologie, Institut national de santé publique du Québec, Québec, Canada

^7^ Institut sur le vieillissement et la participation sociale des aînés, Université Laval, Québec, Canada

^8^ Department of Epidemiology and Community Medicine, Faculty of Medicine, University of Ottawa, Ottawa, Canada

^9^ School of Public Health and Health Systems, and Department of Psychology, University of Waterloo, Waterloo, Canada

^10^ Centre de recherche de l’Institut universitaire en santé mentale de Québec, Québec, Canada

**Table S1**. Characteristics of included (n=669) and excluded (n =507) subjects at study entry

| Characteristics | Included  (with follow-up) (n=669) | Excluded  (no follow-up) (n=507) | P-value |
| --- | --- | --- | --- |
| Age, y | 80.7 ± 6.3 | 84.8 ± 6.5 | <0.001 |
| Sex, female, n (%) | 406 (60.7) | 269 (53.1) | <0.01 |
| Education, y | 9.8 ± 4.1 | 9.1 ± 4.1 | <0.01 |
| BMI, kg/m^2^ | 25.8 ± 4.8 | 24.5 ± 5.0 | <0.001 |
| Plasma lipids, g/L | 6.1 ± 1.8 | 5.6 ± 1.5 | <0.001 |
| ApoE ε4 allele, n (%) | 131 (19.7) | 106 (21.0) | 0.58 |
| Residence area, n (% urban) | 587 (88.4) | 439 (87.1) | 0.51 |
| Smoking, n (% yes)^a^ | 284 (47.1) | 235 (55.4) | <0.01 |
| Alcohol drinking, n (% yes)^b^ | 220 (36.5) | 174 (40.2) | 0.23 |
| Vascular score | 1.2 ± 0.8 | 1.3 ± 0.9 | 0.06 |
| 3MS score | 83.7 ± 11.1 | 76,8 ± 12.5 | <0.001 |
| Plasma OC concentrations (μg/L)^c^ |  |  |  |
| PCB 105 | 0.03 (0.01 – 0.04) | 0.02 (0.01 – 0.04) | 0.40 |
| PCB 118 | 0.14 (0.08 – 0.23) | 0.13 (0.08 – 0.23) | 0.77 |
| PCB 138 | 0.24 (0.17 – 0.35) | 0.25 (0.16 – 0.37) | 0.92 |
| PCB 153 | 0.43 (0.29 – 0.60) | 0.43 (0.29 – 0.62) | 0.55 |
| PCB 156 | 0.06 (0.04 – 0.08) | 0.06 (0.04 – 0.09) | 0.06 |
| PCB 163 | 0.08 (0.05 – 0.11) | 0.08 (0.06 – 0.12) | 0.11 |
| PCB 170 | 0.10 (0.07 – 0.14) | 0.11 (0.08 – 0.15) | 0.05 |
| PCB 180 | 0.34 (0.24 – 0.49) | 0.36 (0.26 – 0.52) | 0.07 |
| PCB 183 | 0.03 (0.02 – 0.05) | 0.03 (0.02 – 0.05) | 0.98 |
| PCB 187 | 0.09 (0.06 – 0.14) | 0.10 (0.06 – 0.15) | 0.31 |
| β -HCH | 0.13 (0.08 – 0.19) | 0.13 (0.08 – 0.19) | 0.81 |
| HCB | 0.17 (0.10 – 0.28) | 0.16 (0.09 – 0.29) | 0.38 |
| Oxychlordane | 0.11 (0.07 – 0.14) | 0.11 (0.08 – 0.15) | 0.51 |
| *cis*-nonachlor | 0.02 (0.01 – 0.03) | 0.02 (0.01 – 0.03) | 0.65 |
| *trans*-nonachlor | 0.14 (0.10 – 0.21) | 0.14 (0.10 – 0.21) | 0.97 |
| *p, p’*-DDT | 0.07 (0.03 – 0.13) | 0.07 (0.03 – 0.13) | 0.52 |
| *p, p’*-DDE | 4.1 (2.1 – 7.7) | 4.4 (1.9 – 8.3) | 0.44 |

Note: 3MS, Modified Mini-Mental State Examination; BMI, body mass index; OC, organochlorine; PCBs, polychlorinated biphenyls ; β-HCH, Beta hexachlorocyclohexane; HCB, hexaclorobenzene; *p, p’*-DDT, 1,1,1-trichloro-2,2-bis(*p*-chlorophenyl)ethane; *p, p’*-DDE, 1,1-dichloro-2,2-bis(*p*-chlorophenyl)ethylene.

^a^Ever been smoking regularly (nearly every day)

^b^Ever been drinking regularly (once a week)

^c^Values are represented as median (interquartile range).

Values are represented as mean ± standard deviation unless stated otherwise.

*P*-values were obtained using χ^2^ test for dichotomous variables and *t*-tests or non-parametric Wilcoxon rank-sum tests for continuous variables, as applicable.

Information was missing on BMI for 35 subjects included and 48 excluded; on APOE ε4 status for 3 subjects included and 2 excluded; on residence area for 5 subjects included and 3 excluded; on smoking status for 66 subjects included and 83 excluded; on alcohol drinking for 66 subjects and 74 excluded; on plasma total lipids 33 subjects included and 2 excluded.

1. Dr. Éric Dewailly passed away after revising the first draft. [↑](#footnote-ref-1)
